# An overview on monkeypox virus: Pathogenesis, transmission, host interaction and therapeutics

**DOI:** 10.3389/fcimb.2023.1076251

**Published:** 2023-02-10

**Authors:** Shailima Rampogu, Yongseong Kim, Seon-Won Kim, Keun Woo Lee

**Affiliations:** ^1^ Department of Bio & Medical Big Data (BK4 Program), Division of Life Sciences, Research Institute of Natural Science (RINS), Gyeongsang National University (GNU), Jinju, Republic of Korea; ^2^ Department of Pharmaceutical Engineering, Kyungnam University, Changwon, Republic of Korea; ^3^ Division of Applied Life Science (BK21 Four), ABC-RLRC, PMBBRC, Gyeongsang National University, Jinju, Republic of Korea

**Keywords:** monkeypox virus, replication, MPV, drugs, viral structure, transmission mode

## Abstract

*Orthopoxvirus* is one of the most notorious genus amongst the *Poxviridae* family. Monkeypox (MP) is a zoonotic disease that has been spreading throughout Africa. The spread is global, and incidence rates are increasing daily. The spread of the virus is rapid due to human-to-human and animals-to-human transmission. World Health Organization (WHO) has declared monkeypox virus (MPV) as a global health emergency. Since treatment options are limited, it is essential to know the modes of transmission and symptoms to stop disease spread. The information from host–virus interactions revealed significantly expressed genes that are important for the progression of the MP infection. In this review, we highlighted the MP virus structure, transmission modes, and available therapeutic options. Furthermore, this review provides insights for the scientific community to extend their research work in this field.

## 1 Introduction to viruses

Viruses are organisms that cause infectious diseases globally and their infections to humans have been observed since ancient times (Chappell and Dermody, 2015). Studies on viruses and viral diseases began in 19^th^ century (Chappell and Dermody, 2015). Reports have shown that at least 219 virus species can cause infections in humans (Woolhouse et al., 2012). The first virus to be identified by Iwanovsky and Beijerinck was the tobacco mosaic virus in 1892 and the foot-and-mouth disease virus by Loeffler and Frosch in 1898 (van Kammen, 1999; Woolhouse et al., 2012; Chappell and Dermody, 2015). In humans, the first discovery was the yellow fever virus in 1901 (Woolhouse et al., 2012; Chappell and Dermody, 2015). Since then, multiple viruses have been discovered.

Several viruses have been infecting humans and posing a challenge in finding effective therapeutics (Wong et al., 2017), such as human immunodeficiency virus (HIV) (Lloyd, 1996; Simon et al., 2006) and SARS-CoV-2 (Dhama et al., 2020; Hu et al., 2021; Rampogu and Lee, 2021a). Particularly, the SARS-CoV-2 has hampered the health system, and this virus also warrants the scientific community to be ready for future pandemics. One such infectious diseases is monkeypox, which has been recently spreading globally.

## 2 Monkeypox

**Figure 1:**
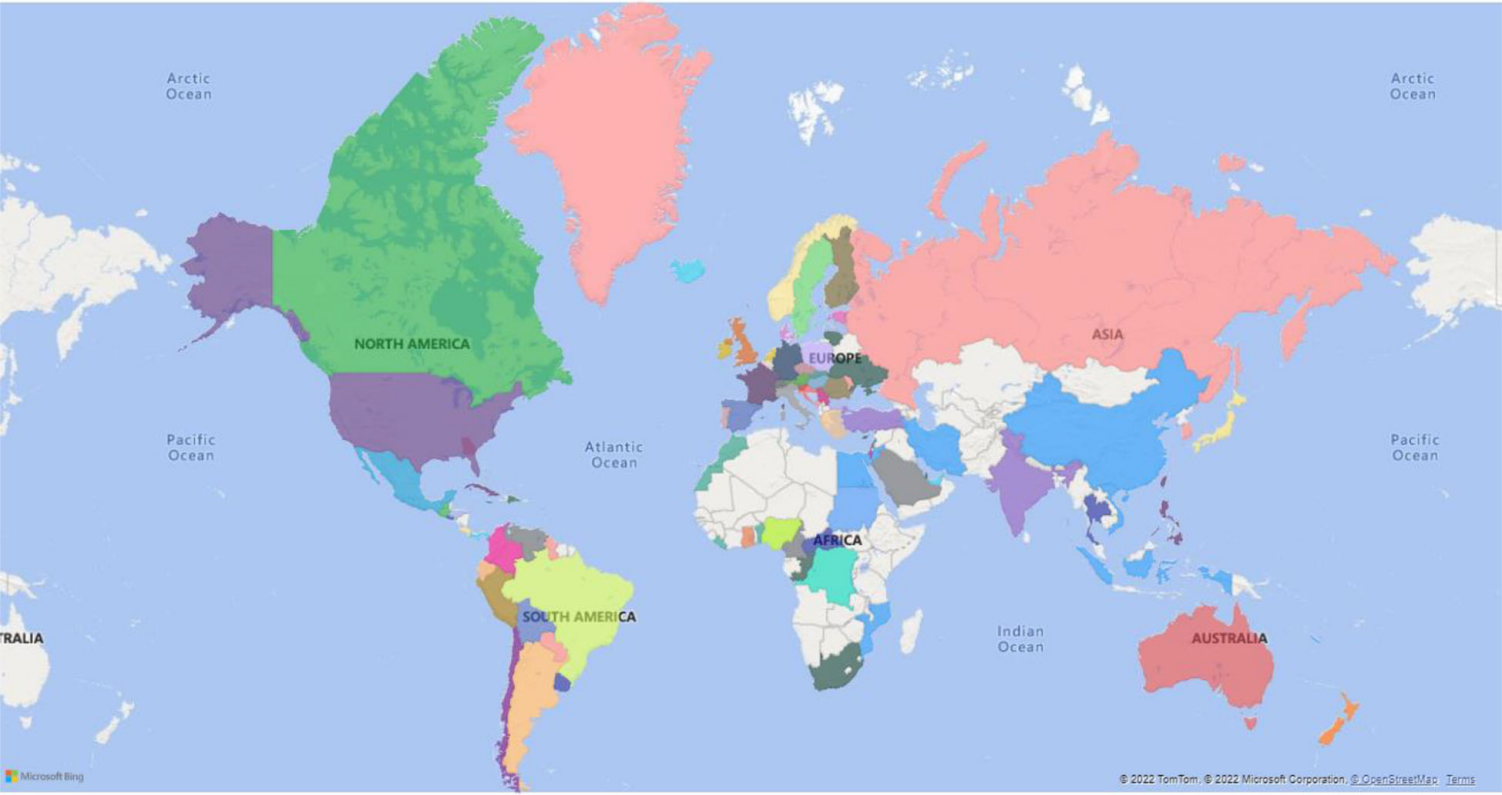
The global spread of monkey pox virus (MPV). The figure is prepared using PowerBI desktop.

Monkeypox (MP) is a zoonotic disease that demonstrates smallpox-like characteristics in humans and is related to variola virus, the causative agent of small pox (Bunge et al., 2022). Monkeypox virus (MPV) belongs to the family: Poxviridae, subfamily: chordopoxvirinae, genus: orthopoxvirus, and species: Monkeypox virus (Moore et al., 2022). Other members of this genus include cowpox, camelpox, and vaccinia (VV) (Pauli et al., 2010; McCollum and Damon, 2014; Silva et al., 2020). As on 14^th^ October 2022, a total of 73,288 cases have been recorded according to https://www.cdc.gov/poxvirus/monkeypox/response/2022/world-map.htm. Distribution of MPV infection in different countries (**Figure 1**).

## 3 Monkeypox and smallpox

Interestingly, variola virus (VARV) and MPV are related antigenically and genetically (Shchelkunov et al., 2001; Cann et al., 2013). However, these viruses vary in the regions of their sequence for virulence and host-range factors at the genome termini (Shchelkunov et al., 2001; Cann et al., 2013). Upon comparing the VARV strain Japan 1951 (Harper, Masterseed) and MPV strains (Zaire 96-I-16), the length of MPV genome was 10,678 bp larger than Variola virus (Shchelkunov et al., 2001; Esposito et al., 2006; Cann et al., 2013). Furthermore, MPV has displayed mutations that influence the process of translation of interferon resistance genes that code for proteins C3L and E3L. The MPV genome also encodes a secreted interleukin (IL)-1β-binding protein, which is absent in VARV genomes. The absence of this vaccinia virus (VACV) - gene corresponds to amplified pathogenicity (Cann et al., 2013).

## 4 History of MPV

The advent of MPV was primarily observed in monkeys shipped from Singapore to Copenhagen in 1958 by von Magnus et al. (Cho and Wenner, 1973; Moore et al., 2022). The name monkey virus is coined after its first discovery from monkeys (Bunge et al., 2022). Approximately, 20-30% of animals have manifested clinical illness (Cho and Wenner, 1973; Moore et al., 2022). Notably, out of the 6 cases in 1970, the first human infection was reported in an infant (9 months old) from the Democratic Republic of the Congo (Ladnyj et al., 1972; Cho and Wenner, 1973; Moore et al., 2022). Four other infections were observed in children from Bouduo, Liberia aged 4-9 years (Cho and Wenner, 1973), and the other case was in a male of 24 years from Sierra Leone (Cho and Wenner, 1973). In 2003, MPV cases were recorded in the USA that is outside Africa (Bunge et al., 2022). Notably, this is caused due to animal-to- human transmission (Reed et al., 2004). Between 2018 and 2021, the MPV spread was observed in the USA, the UK, Israel, and Singapore (Mauldin et al., 2022). By 2022, MPV reached 31 countries (Xiang and White, 2022) with no travel history to endemic countries, and the virus isolates are reported as West African clade (Xiang and White, 2022).

## 5 Genome and structure of MPV

The MPV genome is a linear genome with a size of approximately 197 kb (196,858-base pairs) (Shchelkunov et al., 2001; Alkhalil et al., 2009) comprising ≈190 non-overlapping ORFs >180 nt in length (Kugelman et al., 2014). The central coding region sequence is positioned at ≈56000–120000 nucleotides (Kugelman et al., 2014). This conserved region is flanked by variable ends that comprise inverted terminal repeats (ITRs). The genome encodes biological machinery that is essential for the survival of the virus (Alkhalil et al., 2009). Genes in the central region are necessary for entry, self-replication, and maturation (Alkhalil et al., 2009). The less conserved terminal regions are useful for host-virus interaction (Alkhalil et al., 2009).At the ITR zone, a minimum of 4 ORFs are present (Kugelman et al., 2014).

There are two clades present in the MPV: the Congo Basin strain and West African strain (Kindrachuk et al., 2012). The Congo Basin strain is also known as the Central African strain (ZAI-96). The West African strains are SL-V70, COP-58, and WRAIR-6 (Weaver and Isaacs, 2008). These clades demonstrate dissimilar virulence (Kindrachuk et al., 2012) and are geographically, clinically, and genetically different (Kindrachuk et al., 2012). The Congo Basin strain demonstrated a case fatality rates of approximately 10% noticed in the people who are non-vaccinated than the less lethal West African MPV clade (Kindrachuk et al., 2012). Furthermore, the fatality rate differs with the strain, with 3.6% with the West African clade and 10.6% with the Central African clade (Bunge et al., 2022).

The West African strain is linked with lower transmission within the humans (Hutson et al., 2009), with a difference of 0.55-0.56% nucleotide between both the strains (Chen et al., 2005). A difference of 0.01–0.07% nucleotides is observed among the West African strains (Chen et al., 2005). A recent study conducted through shotgun metagenomics, showed that the MPV belongs to clade 3 and presumably has a single origin (Isidro et al., 2022). Another phylogenetic study was conducted on African monkeypox (Nakazawa et al., 2015). The authors examined fairly large genomic sequence data obtained from the MPV isolates across the area of their distribution to determine the relationship between the clades and among the isolates and further improve the phylogenetic analysis (Nakazawa et al., 2015).

Furthermore, the amplified virulence observed in the Central African strains is believed to be due to D14L (complement inhibitor), D10L (host range protein), B14R (interleukin [IL]-1β binding protein), B10R (apoptotic regulator), and B19R (serine protease inhibitor-like protein) genes (Estep et al., 2011) (Karumathil et al., 2018). The West African strains are devoid of D14L (Estep et al., 2011). The orthologs of D10L and B19R orthologs are conserved in both clades (Chen et al., 2005). The orthologs of ZAI-96, B10R and B14R are absent in West African strain (Chen et al., 2005). Additionally, selective repression of the host response is noticed in Central African strain demonstrated by its ability to regulate apoptosis in the host (Kumar et al., 2022).

**Figure 2:**
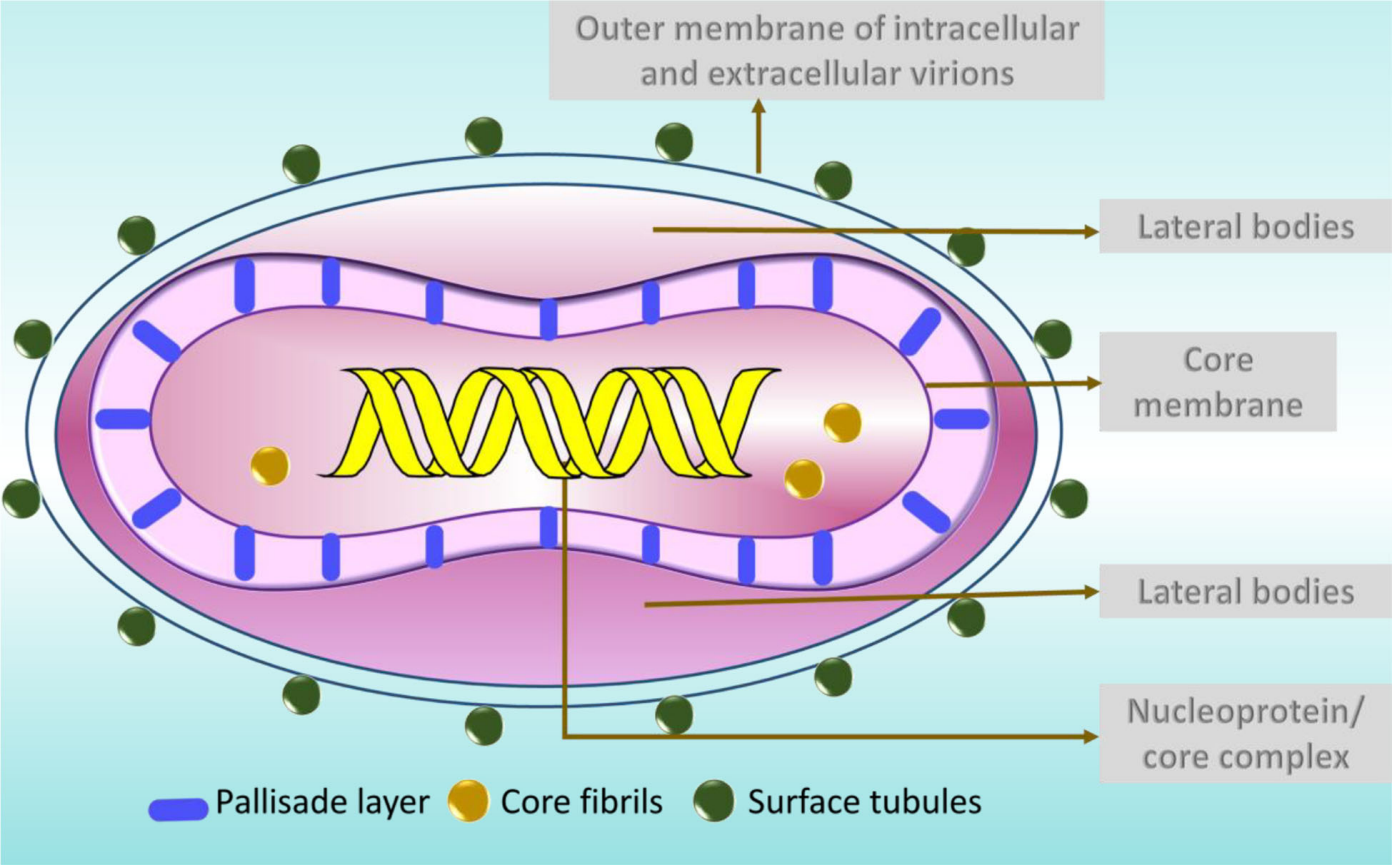
The structure of MPV particle.

MPV is one of the largest and most highly complex viruses (Barreto-Vieira and Barth, 2015) demonstrating a brick-shaped structure with a length of 220 - 450 nm and a width ranging from 140 - 260 nm (Hyun, 2022). It has four components: core, lateral bodies, outer membrane, and outer lipoprotein envelope (Sklenovská, 2020). The core is the central part, encompassed by core fibrils and double stranded viral DNA. This layer is encircled by a rigid structure called the palisade layer (Sklenovská, 2020). The outer membrane accommodates the palisade layer, lateral bodies and the central core (Sklenovská, 2020). The structures called the surface tubules and are present on the outer surface (Sklenovská, 2020) (**Figure 2**).

Typically two types of virions are noticed in the negatively stained preparations of orthopoxviruses, namely the M form (mulberry) and C form (capsule) (Jezek and Fenner, 1988). The M-type virions are undamaged, while the C forms are the damaged ones (Jezek and Fenner, 1988). In C type virions, the stain percolates the cell, exposing the outer membrane and partially exposing the internal structures (Jezek and Fenner, 1988). Clinically, the M-types is present in the vesicle fluid and the C type in dried scabs (Jezek and Fenner, 1988).

## 6 Reservoir and transmission

The common reservoir for MPV is thought to be not only monkeys (Moore et al., 2022), but also other animals such as squirrels (Khodakevich et al., 1987), and sooty mangabey. (Radonić et al., 2014). Although the rate at which the virus resides among animals is still obscure, rodents are thought to be the reservoir hosts for this virus (Nolen et al., 2015; Wilson, 2017). Infections may also occur in mice, rats, humans (Learned et al., 2005; Nolen et al., 2016; Besombes et al., 2019), and prairie dogs (Tesh et al., 2004; Moore et al., 2022). MPV transmission may occur when a healthy individual comes into contact with the skin lesions, droplets, and bodily fluids of infected animals. Partially cooked food might also be a reason for the infection (Ahmed et al., 2022). The infection may also result from to contact with contaminated fomites (Moore et al., 2022). Additionally, the infections may also result due to activities that enhance exposure to animals, such as sleeping on the ground outside and habituating near the forest (Fuller et al., 2011; Guagliardo et al., 2020). The risk of infection may also be noted when eating bushmeat or wild game (Kmiec and Kirchhoff, 2022).

**Figure 3:**
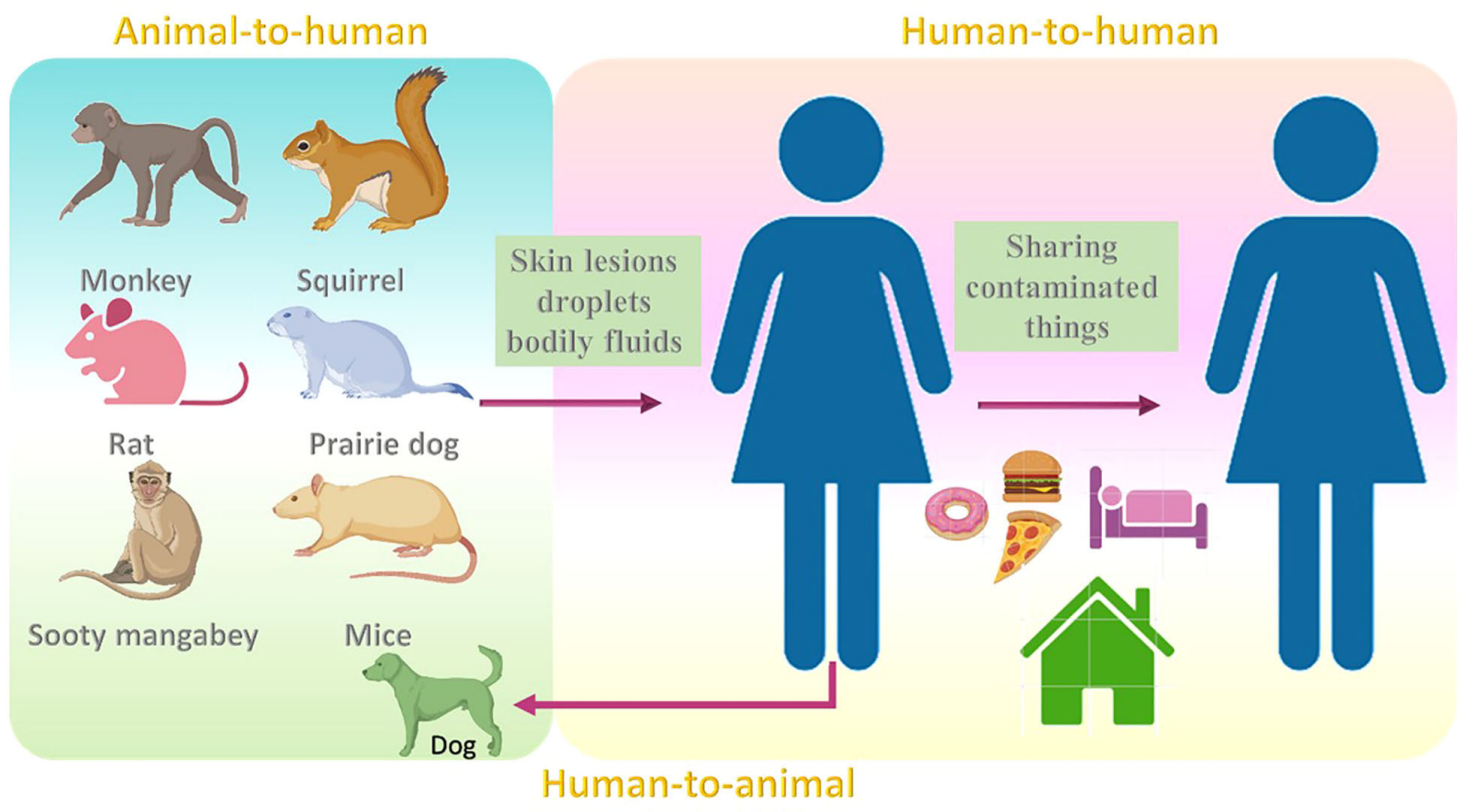
Transmission modes of MPV from animals (reservoirs) to humans and humans to animals.

In 2018, MPV transmission was observed from an infected patient to a healthcare assistant which might be due to contact with bedding that was contaminated (Vaughan et al., 2020). Human-to-human transmission has also been noted in the Democratic Republic of the Congo (Nolen et al., 2016). Transmission within humans can also occur by sharing the same household and consuming food or water from the same dish as the patient (Bunge et al., 2022). In 2022, the United Kingdom witnessed an increase in MPV cases, categorized into three different events (Vivancos et al., 2022). The first case was in Nigerian imports, the second case was a cluster in a household, and the third case was reported in men with no reported link with previous cases or the travel history (Vivancos et al., 2022). One study reported the first case of MPV transmission from a human to dog (Seang et al., 2022). A recent report showed that out of 528 infections across 16 countries, 98% were bisexual or gay, 75% of the infections were seen within the white, and 41% were positive for HIV (Thornhill et al., 2022) (**Figure 3**).

## 7 Replication

To gain insight onto MPV replication, we relied on pox virus replication. Poxvirus replication occurs in the cytoplasm in specific structures known as Guarnieri bodies (Kieser et al., 2020; Kaler et al., 2022). These bodies are also called factories obtained from infecting particles (Kieser et al., 2020). The count (number) of factories depends on the multiplication rate of infection (Moss, 2013). The factory is the site where transcription translation, and assembly of virions take place (Moss, 2013).

Generally, there exists two types of infectious forms of MPV: the mature virion [MV, also called intracellular mature virions (IMV)] and the enveloped virion (EV, also called extracellular enveloped virions (EEVs)) and cell-associated enveloped virions (CEVs) (Chung et al., 2006; Moss, 2012). The MV has a single membrane, and the EV has an additional outer membrane that is cleaved before fusion. When the MV is enclosed within an endosomal membrane or trans-Golgi, it forms a triple-membrane. These are hence termed wrapped virions (WVs, also called intracellular enveloped virions (IEVs) (Moss, 2012). Unenclosed MVs remain free until cell lysis occurs (Chung et al., 2006; Moss, 2012). Both MV and EV are infectious and can spread the disease (Moss, 2012; Sklenovská, 2020). Comparatively, the stable MV transmits the infection between the host animals, while EVs with fragile external membranes are instrumental in spreading the disease within the host (Moss, 2012; Sklenovská, 2020).

The proteins facilitate the attachment of the virus to the cell, fusion of the membrane and entry into the host cell (Kaler et al., 2022). The single membrane in MV and an extra outer membrane in the EV perform the disruption prior to fusion (Kaler et al., 2022). There are a total of four viral proteins connected to the MV that help in the process of attachment of MV to the host cell (Kaler et al., 2022).

**Figure 4:**
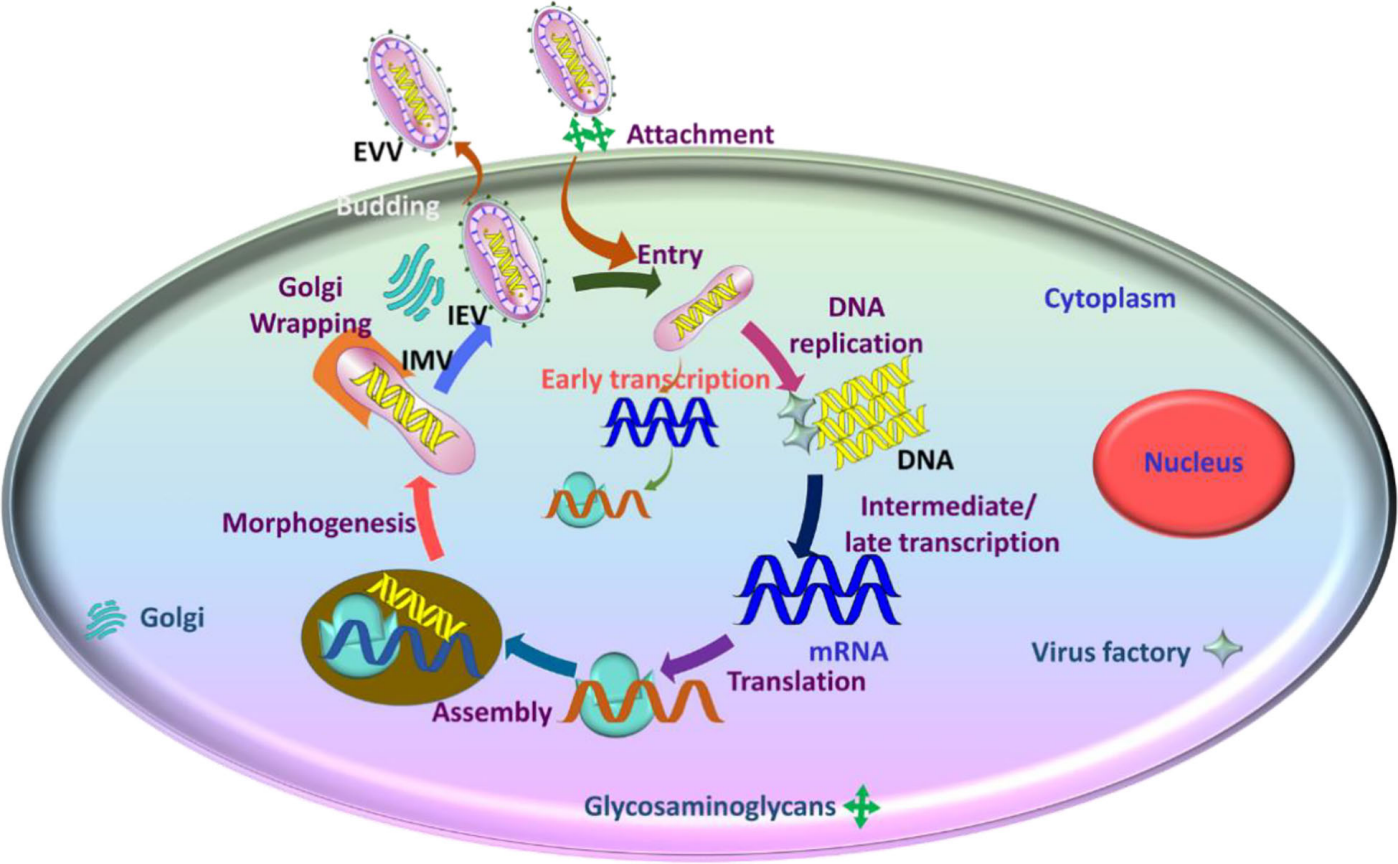
The steps involved in the replication of MPV and the generation of new viral progeny.

The virus attaches to the host *via* 11 to 12 non-glycosylated transmembrane proteins (4- to 43-kDa) (Moss, 2012; Kaler et al., 2022). In some pox viruses, laminin and heparine sulfate aid in the attachment (Kmiec and Kirchhoff, 2022). After infection DNA synthesis is initiated for no more than 2 hours (Moss, 2013). For replication, MV initially uncoats to gain entry into the cytoplasm (Sklenovská, 2020). The early genes are then expressed after the inactivation of the cells defence mechanisms. This inactivation is achieved by the generation of prepackaged viral proteins and the enzymatic factors (Sklenovská, 2020). Subsequently, the early messenger RNA (mRNA) is synthesized through the DNA-dependent RNA polymerase of the virus. The translated early mRNA assists another uncoating mechanism, replication of DNA, and generation of intermediate transcription factors. (Sklenovská, 2020). Then the transcription and translation of intermediate mRNA occurs to promote the late mRNAs expression. Further, the translation of late mRNAs into structural and nonstructural proteins initiates (Sklenovská, 2020). The proteins that are translated are gathered together with the concatemers of DNA that are processed in the earlier step of replication (Sklenovská, 2020). They are enclosed to form an immature virions (IMVs) that transform to MV which are devoid of external membrane and causes infection when liberated due to the disruption of the cell. (Hiller and Weber, 1985; Bray and Buller, 2004; Roberts and Smith, 2008) They then travel into the inner cell membrane aided by the microtubules and eventually fuses to from the cell-associated virions (CEVs). These trigger the actin polymerization and the development of the filaments. The CEVs exists the cell that are now called the extracellular enveloped virions (EEVs) (Roberts and Smith, 2008) (**Figure 4**).

## 8 Symptoms and clinical manifestations of MP

The symptoms of MP are similar to those of smallpox but are generally milder (Ligon, 2004). Once exposed to an infection, the incubation period is between 10 and 14 days and followed by a prodromal period of two days (Weaver and Isaacs, 2008). The individuals demonstrate, muscle aches, backache, headache, intense asthenia, fever between 38.5°C and 40.5°C (McCollum and Damon, 2014) with swollen lymph nodes. Often, patients experience discomfort and exhaustion (Ligon, 2004). The feature that separates monkeypox from smallpox is lymphadenopathy (Moore et al., 2022). Mucosal lesions are observed in the mouth (enanthem) (Kaler et al., 2022) after 1 or 2 days. The skin lesions appear on the face and extremities. According to the World Health Organization (WHO) the face is highly affected, as noticed in 95% of the cases. In 75% of the cases, the palms and soles are affected and in 75% of the cases the oral mucous membranes are affected. The affect is also noticed on genitalia and conjunctivae in 30% and 20% of the cases.

The lesions progress through four stages namely, the macular (development of macular lesions), popular (the lesions are a slightly raised), vesicular (the lesions are raised clearly and filled with fluid), and pustula (lesions are filled with opaque fluid and forms a depression at the Centre and remain for about 5 - 7 days) in the next 2 - 4 weeks (Moore et al., 2022). After the pustular phase, the formation of the crust starts and desquamates in the next 7 - 14 day (Moore et al., 2022). Once the crusts are fallen off, the patients are no longer infectious (Moore et al., 2022). Lesions can cause dyspigmented scars in a few instances (Weaver and Isaacs, 2008).

**Figure 5:**
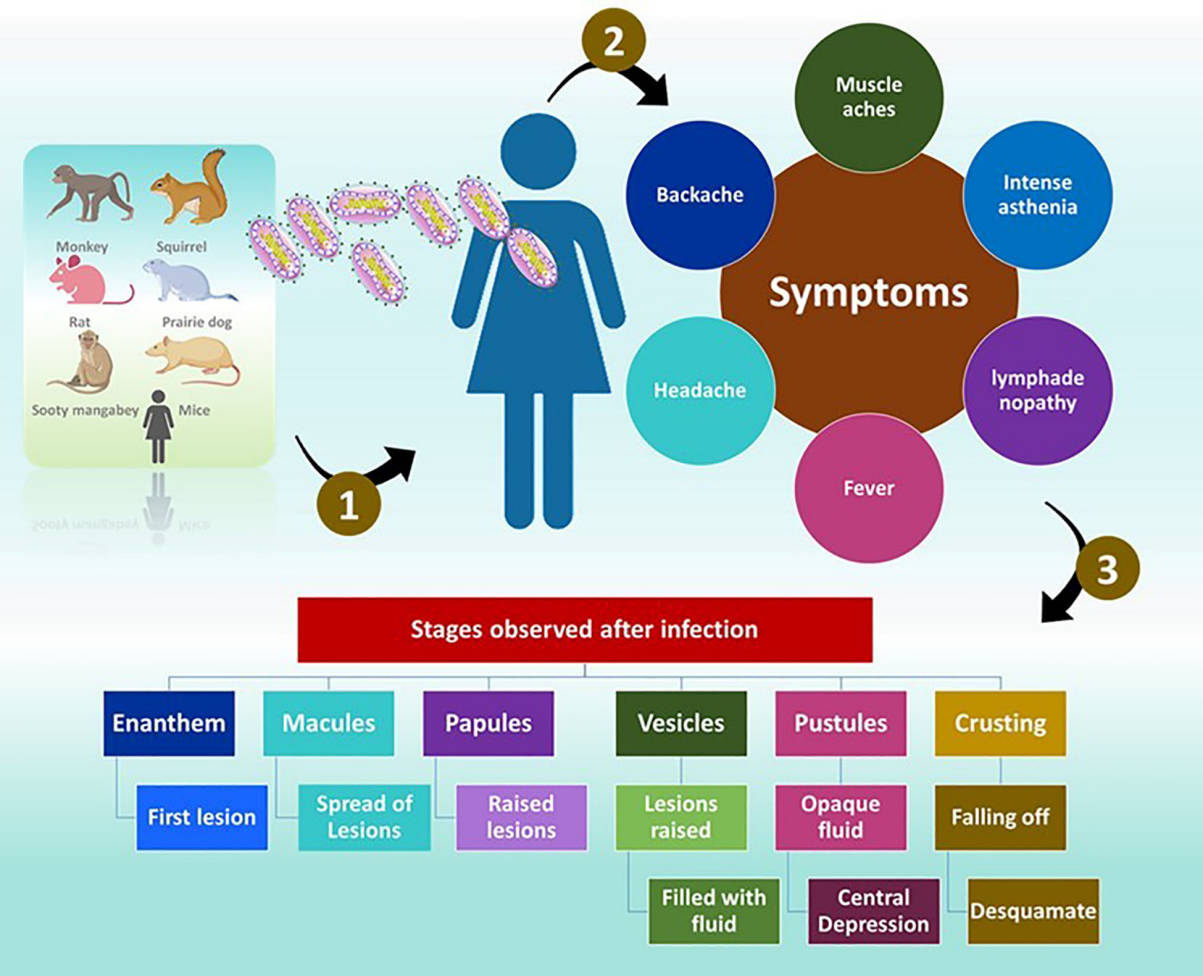
Infection with MPV. The symptoms and different stages observed in an infected individual.

The distribution of the lesion is typically centrifugal and appears firm, deep, well-circumscribed and umbilicated (McCollum and Damon, 2014). Additionally, dissimilarities were observed in the morphology of lesions between vaccinated and unvaccinated people (McCollum and Damon, 2014). In individuals those who were vaccinated <20 years before the infection, the lesions were smaller and fewer, with the centrifugal spreading of the rash (McCollum and Damon, 2014). The skin of patients is firm, inflamed, and painful before the formation of crusts (McCollum and Damon, 2014) (**Figure 5**).

Sequelae and serious complications have also been noticed usually in unvaccinated individuals of approximately 74% compared to those in the vaccinated individuals (39.5%) (McCollum and Damon, 2014). Patients develop pulmonary distress or bronchopneumonia indicating that an infection of the lungs could be a secondary infection (McCollum and Damon, 2014). In others, vomiting or diarrhoea leading to acute dehydration was noticed (McCollum and Damon, 2014). Encephalitis, ocular infections and septicaemia were also recorded, with an average case-fatality rate of 11% in unvaccinated patients with children being highly vulnerable (McCollum and Damon, 2014).

## 9 Virus-host interaction

It has been reported that the MPV encompasses a substantial number of accessory genes and notably hasa broader host range (Bonilla-Aldana and Rodriguez-Morales, 2022; Xiang and White, 2022). Interestingly, the loss of an accessory gene in approximately 17% of samples from West African strain demonstrated an association with an upsurge in human-to-human transmission (Xiang and White, 2022).

A gene-expression profile study was conducted to understand the viral-host biology (Alkhalil et al., 2010). The experiment was conducted on kidney epithelial cells (MK2) of Macaca mulatta using the GeneChip rhesus macaque genome microarrays (Alkhalil et al., 2010). The findings from this study demonstrate the function of ion channels, cell cycle regulators, histones, and actin play a role in MPV infection (Alkhalil et al., 2010).

A recent bioinformatics study based on transcriptome analysis was conducted to identify biomarkers and signaling pathways in cells infected with monkeypox (Tang et al., 2022). GSE36854 and GSE11234 were retrieved from Gene Expression Omnibus (GEO) (Tang et al., 2022). The results showed that from the GSE36854 dataset, 84 genes were significantly different. The protein-protein interaction (PPI) interactions and the identification of hub genes has revealed that the genes such as ZC3H12A, IER3, EREG, IFIT2, AREG, IL11, IFIT1 IER2, NFKBIE, and FST as the 10 hub genes. (Tang et al., 2022). The genes IFIT1 and IFIT2 (antiviral genes) were noticed to be remarkably repressed. Furthermore, upon searching the drugs that essentially target the hub genes, itraconazole and AP-26113 were found to stimulate the expression levels of IFIT1 and IFIT2 illuminating their ability as MP therapeutics (Tang et al., 2022).

To discover variations in the expression of genes, co-regulated genes, and the pathways that are of concern during MP progression, another research group worked on *in vitro* models that were infected with MPV (Xuan et al., 2022). The two models on which several analysis were performed were, rhesus monkey (*Macaca mulatta*) kidney epithelial (MK2) cells and human immortal epithelial cancer (HeLa) cells (Xuan et al., 2022). The results showed that in the animal cell line model, the prominent regulators were histamine, plasmin and cluster of differentiation 40 (CD40), whereas in the human cell line model, the neutrophil-related signaling pathways and macrophages were noted (Xuan et al., 2022). Additionally, in both models, certain genes that were remarkably expressed during the progression of the infection including, TNFAIP3, IL11, ADORA2A, DUOX1, BIRC3, PTX3, CXCL1, LIF, IER3, IL6, ZC3H12A, EGR1, CSF2, and CCL2. Additionally, epigenetic regulators were also observed, namely, HIST1H3D and HIST1H2BJ (Xuan et al., 2022). Furthermore, a computational study uncovered the over-expression of some histones in both humans and monkeys infected with monkeypox-infected cells (Xuan et al., 2022). The elevated histone members are HIST1H2BB, HIST1H2AK, HIST2H2AB, HIST1H2AC, HIST1H2BM, HIST1H2BJ, HIST1H2BH, HIST1H2AD, HIST1H3D, and HIST1H1B highlighting the possibility of the role epigenetic regulators in monkeypox infection (Xuan et al., 2022).

Another study also has identified the upregulation of histone genes namely, HIST1H2BJ, HIST4H4, HIST1H3I, HIST1H2AD, and, HIST1H1D, while H1F0, the linker histone was downregulated (Alkhalil et al., 2010). Certain genes such as MYCBP2, PRMT3, RARS2, FBXO11 and MYST2 were repressed (Alkhalil et al., 2010). During the infection, the repression of ten ion channels and transporters was observed. The cell cycle regulators and actin MPV infection were noticed to participate in MPV infection (Alkhalil et al., 2010). Furthermore, the disease biomarkers can be discovered through proteomics analysis (Wang et al., 2022).

Bourquain et al, conducted an experiment to determine how poxviruses vary their host cell gene expression. HeLa cells were selected, and the changes were recorded using microarrays that are representative of the whole human genome (Bourquain et al., 2013). HeLa cells were treated with CPXV Brighton Red (BR) reference strain, central African MPV strain MSF-6, or the mouse-pathogenic VACV strain IHD-W (Bourquain et al., 2013). In particular to the MPV results, merely 321 (1.1%) transcripts showed 2-fold expression variations with 219 (68.2%) upregulated transcripts and 102 downregulated transcripts (Bourquain et al., 2013). Interestingly, the results also showed common transcripts among infections (Bourquain et al., 2013). The MPV has regulated 321 host transcripts of which 241 (75.1%) were observed with cowpox virus (CPXV) and 148 (46.1%) within VACV infection (Bourquain et al., 2013). Genes such as DUSP5/6, SPRED1/2, and SPRY2/4 are upregulated along with EGR1 and EGR2. After infection, the EGR1 upregulation was observed *via* the MAPK-ERK pathway (Bourquain et al., 2013).

In MPV infection, the genes that take part in the negative regulation of MAPK activity and intracellular protein kinase cascade were enriched, and they induced genes associated with chemotaxis or leukocyte activation (Bourquain et al., 2013). Hammarlund et al, reported that monocytes infected with MPV do not recognize antiviral CD4^+^ and CD8^+^ T Cells (Hammarlund et al., 2008). MPV can cause infection in primary human monocytes without eliciting the production of inflammatory cytokines (IFNγ or TNFα) (Hammarlund et al., 2008). It was also reported that MPV impedes the activation of T- cell by VV, implying that MPV bears an immunomodulatory protein which is absent in VV (Hammarlund et al., 2008). This inhibition *in trans* can also obstruct the activation of T - cells by other virus-infected cells that are not precisely MPV-infected, suggesting that MPV is immunosuppressive and immune-evasive (Hammarlund et al., 2008). Cell-associated factor/factors generated in MPV hinder the activation of T - cells autonomously by MHC class I or class II processing or presentation (Hammarlund et al., 2008).

## 10 Treatments against MPV

### 10.1 Small molecule inhibitors

The replication of MPV can be brought about by the RNA interference using genome-wide expression studies combined with a bioinformatics approach (Alkhalil et al., 2009). Accordingly 12 viral genes were targeted using small interfering RNA (siRNAs). Eventually, siA6-a inhibited replication of the virus for 7 days when administered at 10 nM (Alkhalil et al., 2009). Additionally, this study also demonstrated the significance of the A6R gene in replication (Alkhalil et al., 2009). The use of interferon-β (IFN-β) is another approach to curb MPV spread and multiplication (Johnston et al., 2012). IFN-β has been approved by the FDA for treating multiple sclerosis (Johnston et al., 2012). IFN-β administration after 6-8 hours after infection remarkably inhibited the production and spread of MPV, thereby highlighting the potential of IFN-β as an effective therapeutic option against MPV (Johnston et al., 2012). In previous studies, silver-based nanoparticles have been found to repress plaque formation of MPV with a diameter of 10 nm (Rogers et al., 2008), suggesting the role of silver-based nanoparticles as MPV treatment option (Rogers et al., 2008).

Tecovirimat may offer improved survival when administered for up to 8 days after the lethal aerosol MPV challenge in cynomolgus macaques (Russo et al., 2018). It offers a shield from the clinical effects of the disease earlier than 5 days after the challenge (Russo et al., 2018). This FDA approved drug is used to treat smallpox (Smith et al., 2011; Grosenbach et al., 2018; Laudisoit et al., 2018; Russo et al., 2018). The compound tecovirimat is believed to repress the product of F13L gene which is present across orthopoxviruses (Matias et al., 2022) and is used for orthopoxvirus wrapping (Duraffour et al., 2008; Duraffour et al., 2015). The drug brincidofovir, a nucleotide analogue, has demonstrated promising results in animal models when assessed against MPV (Adler et al., 2022). Improved survival was observed in prairie dog models, administered with brincidofovir soon after monkeypox contact (Hutson et al., 2021; Siegrist and Sassine, 2022).

Computational methods are paramount in the development and design of new drugs. The predominant methods include molecular docking (Morris and Lim-Wilby, 2008; Rampogu and Lee, 2021c; Rampogu and Lee, 2021a), and molecular dynamics simulation (Durrant and McCammon, 2011; Rampogu and Lee, 2021a). Pharmacophore modeling can also be adapted when there are any experimentally known ligands/inhibitors or when the X-ray structure of the target protein is present (Yang, 2010; Rampogu et al., 2020). Homology modeling can be used to build a structure when the X-ray structure is not resolved (Muhammed and Aki-Yalcin, 2019). In one study, five targets were investigated, and compounds, such as NMCT, rutaecarpine, nilotinib, simeprevir, hypericin, naldemedine, fosdagrocorat and lixivaptan (Lam et al., 2022), were found to have potential inhibitory activities. These five targets were A48R, A50R, D13L, F13L and I7L (Lam et al., 2022). An *in silico* study revealed that the compound fludarabine is a potential inhibitor of the MPV target DNA-dependent RNA polymerase subunit (A6R) along with two other targets, protein catalysing the envelopment of intracellular mature virus particles (F13L) and proteins involved in cell entry (D8L) (Altayb, 2022). Sahoo et al., identified four potential inhibitors, Tipranavir, Cefiderocol, Doxorubicin, and Dolutegravir towards the targets thymidylate kinase and D9 (decapping enzyme) (Sahoo et al., 2022) (**Table 1**).

**Table 1:** Current treatments/small molecules available for MPV therapeutics.

Inhibitor	Method	Acts on	Dosage	Reference
siA6-a	genome-wide expression, bioinformatics	A6R	10 nM	(Alkhalil et al., 2009)
IFN-β	*in vitro*	MPV spread and multiplication	–	(Johnston et al., 2012)
NMCT, Rutaecarpine, Nilotinib, Simeprevir, Hypericin, Naldemedine, Fosdagrocorat and Lixivaptan	Computational	A48R, A50R, D13L, F13L, I7L	–	(Lam et al., 2022)
Fludarabine	Computational	A6R	–	(Altayb, 2022)
Tipranavir, Cefiderocol, Doxorubicin, and Dolutegravir	Computational	Thymidylate kinase and D9	–	(Sahoo et al., 2022)
Tecovirimat	FDA-approved drug for smallpox	F13L	–	(Matias et al., 2022)
Brincidofovir	FDA-approved drug for smallpox	The effect shown in prairie dogs		(Adler et al., 2022)
Vaccinia immune globulin (VIG)	FDA-approved	intramuscular	–	(Siegrist et al., 2020)
CRISPR/Cas9	vaccinia virus (VACV) model	A17L, E3L, and I2L	–	(Siegrist et al., 2020)

A study was conducted to evaluate the role of specific genes in viral replication and pathogenicity (Lopera et al., 2015). Correspondingly, a bioinformatics approach has been adapted to discover genomic regions in MPV possessing numerous virulence genes (Lopera et al., 2015). Following this, two regions were selected, and the study was conducted *in vitro* and *in vivo* after single deletion and double deletion of the selected genes. The results demonstrated that simultaneous deletion of both genes led to a decrease in replication observed in cell culture (Lopera et al., 2015). When either region was deleted, a remarkable amplified attenuation *in vivo* was observed (Lopera et al., 2015).

### 10.2 Vaccination

In addition to small molecules, vaccines are also widely used as a treatment option (Hooper et al., 2004). Notably, the smallpox vaccine has demonstrated protection in Nonhuman Primates against MPV (Hooper et al., 2004). It is reported that smallpox has provided approximately 85% protection against monkeypox (Bunge et al., 2022). JYNNEOS is a modified vaccinia ankara (MVA) vaccine that has been approved for MPV disease in adults ≥18 years of age (Rao et al., 2022). This was a subcutaneous injection administered at two doses with 28 days apart (Rao et al., 2022). In 2017, healthcare individuals were administered with IMVAMUNE^®^ which is a smallpox vaccine (Petersen et al., 2019). This study aimed to understand the immunogenicity, effectiveness, and safety of IMVAMUNE^®^ in patients at high risk for MPV (Petersen et al., 2019). Another smallpox vaccine, the live VACV Dryvax protects against monkeypox (Heraud et al., 2006; Earl et al., 2008). A multivalent DNA vaccine with eight VACVs virus (Western Reserve strain genes: A4L, A27L, A33R, A56R, B5R, F9L, H3L, and L1R) confers protection against MPV in nonhuman primates *cynomolgus macaques* (Hirao et al., 2011).

It has been observed that, patients vaccinated for smallpox showed immunity (orthopoxvirus [OPXV], IgG and memory B cells) upon exposure to MPV (Nguyen et al., 2021). Interestingly, the smallpox vaccine triggers both humoral and cell-mediated responses towards OPXV which also includes MPV (Nguyen et al., 2021). They target a host of viral elements, thereby hindering viral replication (Nguyen et al., 2021). Vaccinia immune globulin is an approved medication used after contacting the MPV, which is administered intramuscularly (Lederman et al., 2012; Siegrist et al., 2020; Parker et al., 2021). In one study, CRISPR/Cas9 was used to treat orthopoxviruses with VACV, which was used as a model organism (Siegrist et al., 2020). This study focused on the indispensable conserved genes A17L, E3L, and I2L using an adeno-associated virus as a vector (Siegrist et al., 2020). The results have shown a reduction in the viral titre further protecting host cells (Siegrist et al., 2020). An assay based on CRISPR-Cas12a and real-time PCR was used to detect MPV (Li et al., 2006; Sui et al., 2022).

In order to find effective therapeutics, few clinical trials have been conducted. Upon searching for clinical trial information, it was found that nine studies are currently being performed that are either on-going or completed (https://clinicaltrials.gov/)

## 11 Conclusion and future outlook

Viruses are notorious microorganisms that cause serious human infections. Pox viruses are not new as they exist in reptiles, birds, insects, and mammals. Hence, they are also known as ancient viruses (Alakunle et al., 2020). Human MP is a zoonotic disease that is recently spreading globally. In order to prevent the spread of the disease, the contact tracing is an important step (Titanji et al., 2022). If a person is in close proximity with a confirmed case of monkeypox, then that individual should be observed for development of symptoms for 21 days (Titanji et al., 2022).

Since the treatment options for monkeypox are limited (Kaler et al., 2022), the available research methods, such as computational drug discovery, could be an effective method to identify drugs (Ou-Yang et al., 2012; Sliwoski et al., 2014). This approach is useful for discovering new drugs over a short period (Leelananda and Lindert, 2016). Drug repurposing is another approach that can be used to identify mmediate candidate compounds (Pushpakom et al., 2018; Begley et al., 2021). This approach has been proven to be promising for the treatment of SARS-CoV-2. Remdesivir is one such candidate (Li and Peng, 2021). To prevent the spread of virus in the developing countries the awareness on health hygiene is essentially important as the samples from the excreta are reported to have monkeypox virus DNA (Singla and Shen, 2022). Additionally, the knowledge and treat from the pandemics and similar kind of diseases should be widely spread among all the sections of the people (Poland et al., 2022). Countries need to review their approach and preventive measure are to be taken during the outbreaks and be prepared for any such kind in the future (Poland et al., 2022). To conclude, since the spread of MPV is swift, it demands extra awareness and thoughtfulness to control the transmission and thereby to mitigate the disease and further its future reappearance.

## Author contributions

SR and KW conceived the idea. SR wrote the manuscript. KW, S-WK, and YK reviewed the manuscript and revised. All authors contributed to the article and approved the submitted version.
